# LEF1 isoforms regulate cellular senescence and aging

**DOI:** 10.1111/acel.14024

**Published:** 2023-11-13

**Authors:** Minxue Jia, Khaled Sayed, Maria G. Kapetanaki, William Dion, Lorena Rosas, Saad Irfan, Eleanor Valenzi, Ana L. Mora, Robert A. Lafyatis, Mauricio Rojas, Bokai Zhu, Panayiotis V. Benos

**Affiliations:** ^1^ Department of Computational and Systems Biology University of Pittsburgh Pittsburgh Pennsylvania USA; ^2^ Joint Carnegie Mellon University‐University of Pittsburgh Ph.D. Program in Computational Biology Pittsburgh Pennsylvania USA; ^3^ Department of Electrical & Computer Engineering and Computer Science University of New Haven West Haven Connecticut USA; ^4^ Department of Epidemiology University of Florida Gainesville Florida USA; ^5^ Aging Institute of UPMC University of Pittsburgh Pittsburgh Pennsylvania USA; ^6^ Division of Pulmonary, Critical Care and Sleep Medicine, Department of Internal Medicine The Ohio State University Columbus Ohio USA; ^7^ Department of Rheumatology University of Pittsburgh Pittsburgh Pennsylvania USA; ^8^ Department of Medicine University of Pittsburgh Pittsburgh Pennsylvania USA; ^9^ Pittsburgh Liver Research Center University of Pittsburgh Pittsburgh Pennsylvania USA

**Keywords:** aging, cellular senescence, IPF, LEF1, lung

## Abstract

The study of aging and its mechanisms, such as cellular senescence, has provided valuable insights into age‐related pathologies, thus contributing to their prevention and treatment. The current abundance of high‐throughput data combined with the surge of robust analysis algorithms has facilitated novel ways of identifying underlying pathways that may drive these pathologies. For the purpose of identifying key regulators of lung aging, we performed comparative analyses of transcriptional profiles of aged versus young human subjects and mice, focusing on the common age‐related changes in the transcriptional regulation in lung macrophages, T cells, and B immune cells. Importantly, we validated our findings in cell culture assays and human lung samples. Our analysis identified lymphoid enhancer binding factor 1 (LEF1) as an important age‐associated regulator of gene expression in all three cell types across different tissues and species. Follow‐up experiments showed that the differential expression of long and short LEF1 isoforms is a key regulatory mechanism of cellular senescence. Further examination of lung tissue from patients with idiopathic pulmonary fibrosis, an age‐related disease with strong ties to cellular senescence, revealed a stark dysregulation of LEF1. Collectively, our results suggest that LEF1 is a key factor of aging, and its differential regulation is associated with human and murine cellular senescence.

## Graphical abstract

Lymphoid enhancer‐binding factor 1 (LEF1) regulon is a common mechanism of aging in human and murine lungs and in human blood. Experimental evidence supports a direct functional role of the long LEF1 isoform in partially reversing cellular senescence through a possible new regulatory mechanism. LEF1 expression and regulon activity in IPF suggest a diverse mode of action during the early and late stages of the disease.

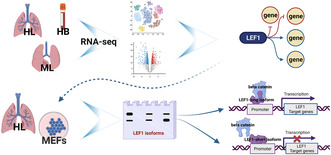

**Abbreviations**
DGE: differential gene expression
IPF: idiopathic pulmonary fibrosis
LEF1: lymphoid enhancer binding factor 1
scRNA‐seq: single cell RNA sequencing

## 1 INTRODUCTION

It is generally accepted that as the world's population is getting older due to the increase in life expectancy (Lauren Medina; Shannon Sabo; Jonathan Vespa; U.S. Census Bureau, 2020), the need of preventing and addressing age‐associated pathologies will also increase. Therefore, it is necessary to heighten our effort in understanding the complex mechanisms underlying healthy aging as opposed to disease‐ridden survival. On the molecular and cellular level there are 12 identified hallmarks of aging (López‐Otín et al., 2023), which form a framework for age‐related research. Cellular senescence, identified as one of the hallmarks of aging, is contributing to aging either through the accumulation of damaged senescent cells or the secretion of pro‐inflammatory cytokines and matrix metalloproteases, often referred to as senescence associated secretory phenotype (SASP) (Di Micco et al., 2021; Rodier & Campisi, 2011). Cumulative evidence supports the existence of rather ubiquitous age‐associated molecular mechanisms which can occur in multiple tissues/organs of the same organism or across different species (Barth et al., 2019). Research on multiple organs and organisms suggests that addressing the underlying mechanisms of aging could impact the course of several pathologies that are currently associated with old age.

While common mechanisms may underlie the process of aging in different organs, the unique physiology of each one of them as well as environmental stressors will also determine how aging impacts their fitness. The lung, as an organ, presents a unique system due to its cellular complexity of at least 40 discrete cell types (Franks et al., 2008) with a large interface surface that is constantly challenged by diverse stressors. Advanced age is a significant risk factor for several lung diseases including COPD (MacNee, 2016) and IPF (Mora et al., 2017) which interestingly present some of the same irregularities in cellular mechanisms that are considered hallmarks of aging, including cellular senescence (Cho & Stout‐Delgado, 2020; De Man et al., 2023; Han et al., 2023). Due to these similarities, the investigation of age‐related molecular changes could lead to a better understanding of disease pathogenesis and eventually more efficient therapeutic interventions (Jia, Agudelo Garcia, et al., 2023; Karampitsakos et al., 2023).

In recent years, the abundance of molecular high‐throughput data has supported the development of a plethora of functional and mechanistic computational analysis tools which are widely used to analyze gene expression data. Importantly, many of these transcription factor (TF) and pathway analysis methods are successfully adapted for single cell RNA‐seq (scRNA‐seq) data (Holland, Szalai, & Saez‐Rodriguez, 2020) and can provide meaningful insights into single cell biology (Jia, Rosas, et al., 2023).

In the current work, we analyzed scRNA‐seq and bulk RNA‐seq data from human lung and blood samples of healthy donors as well as mouse lung samples, focusing on macrophages, T cells, and B cells which were abundant and well‐identified in all datasets. Interestingly, we did not find any differentially expressed genes (young vs. aged) shared between human and murine immune cells. However, when we examined the TF activity (regulons), we were able to identify lymphoid enhancer‐binding factor 1 (LEF1) regulon as a common mechanism of aging in human and murine lungs and in human blood. More importantly, we provide experimental evidence to support a direct functional role of the long LEF1 isoform in partially reversing cellular senescence through a possible new regulatory mechanism.

## 2 MATERIALS AND METHODS

### 2.1 Datasets

For this project we used four publicly available datasets. (1) *GSE128033*, which includes human scRNA‐seq samples taken from whole lung tissue of three young (age ≤ 23, 14,704 cells), two heathy aged (age ≥ 55, 8573 cells), and six IPF donors (age ≥ 69, 26,538 cells). This dataset was generated by our group (Cruz et al., 2021; Morse et al., 2019). (2) *GSE122960*, which includes human scRNA‐seq samples of whole lung tissues of two young (15,770 cells) and three aged (21,981 cells) healthy donors (Reyfman et al., 2019). (3) *GSE124872*, which includes mouse scRNA‐seq samples from whole lung tissue of seven 3‐month‐old (7672 cells) and eight 24‐month‐old mice (7141 cells) (Angelidis et al., 2019). (4) *GSE158699*, which includes bulk RNA‐seq data from blood samples from former and current smokers of the COPDGene study (Regan et al., 2011). This dataset was divided in two groups: young (*n* = 79, age ≤ 55) and aged (*n* = 123, age ≥ 75).

For human scRNA‐seq data, we used all samples (never‐smokers and former smokers; *n* = 10, 60,922 cells) to identify main cell types via unsupervised clustering (package: Seurat; Stuart et al., 2019). Subsequently, we took special care to select healthy samples that are free of smoking‐related or other pathologies by carefully examining the provided records of smoking status and lung histopathology. We did so to ensure that the identified signals can be attributed to aging and not any aging confounders. This process eliminated other publicly available datasets we considered and resulted in the exclusion of two samples from the above datasets. Donor 4 was a former smoker with abnormal cell type distribution (Figure S2); and Donor 2 was excluded due to the histopathology that indicated previous smoking or pollution exposure (Figure S3). Complete list of samples used in cell type identification and presented in Table S1.

### 2.2 Digital cytometry

We used CIBERSORTx (Newman et al., 2019) to infer cell‐type proportions and cell‐specific gene expression from the blood‐derived bulk RNA‐seq data. Deconvoluted cells with an imputed number of genes <5% of the total number in the bulk RNA‐seq dataset were excluded from our analysis. Only CD4^+^‐naïve T cells had an imputed number of genes >5%.

### 2.3 Differential gene expression data analysis

We used Seurat V3 (Stuart et al., 2019) on the human and mouse scRNA‐seq data to determine differential gene expression between aged and young lungs. For the Mouse Lung dataset (ML), we first created a Seurat object for the raw counts using CreateSeuratObject. Then, we calculated the percentage of the mitochondria genes (percent.mt; function: PercentageFeatureSet). Cells with >5% mitochondrial genes (variable: percent.mt) and with number of features <250 or >2000 (variable: nFeatures_RNA) were filtered out. The filtered data were then normalized and scaled (functions: NormalizeData and ScaleData). We also used FindVariableFeatures to find the top 2000 high variable genes. For the Human Lung dataset (HL), we filtered out cells with high percentage of mitochondria genes (percent.mt >35%) and with number of features less than 200 (nFeatures_RNA). Besides, we filtered out empty droplet and doublets identified by Scrublet and emptyDrops (Lun et al., 2019; Wolock et al., 2019). The filtered (missing) data were then imputed using SAVER (Huang et al., 2018). The imputed data were normalized and scaled using the sctransform function, and the number of UMIs per cell as well as the percentage of mitochondrial gene content were regressed out.

In order to create clusters for the different cell types, we performed Principal Component Analysis (PCA) to reduce the dimensionality of the data (function: RunPCA). The FindNeighbors function was then used to construct a shared nearest neighbor (SNN) graph on the dimensionally reduced data from the first 10 principal components. For Human Lung dataset, we further removed the effect from technical or biological confounders using Harmony, and SSN graph was constructed using corrected PCA embeddings (Korsunsky et al., 2019). The FindClusters function was utilized to identify the cell clusters. Each cluster was identified by differentially expressed genomic signatures or automated cell type annotation. For automated cell type annotation, we used the scCATCH package (Shao et al., 2020) where the findmarkergenes function is applied with *p*‐value threshold of 0.05 and logFC threshold of 0.25 to find marker genes for each cluster and then the scCATCH function is utilized to identify the corresponding cell types. To perform DGE analysis between the aged and young cells, we subset the Seurat object by cell type and applied the FindMarkers function with ident.1 = “old” and ident.2 = “young” and the default parameters.

For the bulk RNA‐seq data from the human blood, we used the *limma* R‐package (Ritchie et al., 2015) to regress out the effect of gender and smoking status of the selected subjects from the COPDGene dataset. The differentially expressed genes were defined as the genes with FDR adjusted *p* < 0.05 and abs (log_2−_fold change) >0.25 for all datasets.

### 2.4 Regulon activity

The set of TFs and their transcriptional targets (i.e., regulons) used for the analysis of the human and mouse lung datasets were defined using DoRothEA (Garcia‐Alonso et al., 2019; Holland, Szalai, et al., 2020; Holland, Tanevski, et al., 2020). Each regulon is considered as a gene set and converted into a GeneSet object using the GeneSet() function from the GSEABase package (Morgan et al., 2021). We used the Escape package (Borcherding et al., 2021) to calculate Single Sample Gene Set Enrichment (ssGSEA) scores for each regulon using the enrichIt() function. The ssGSEA scores were calculated for the young and old cells in each cell type separately and the differential ssGSEA score was calculated by subtracting the average score of the young cells from the average score of the old cells (i.e., *diff_score = avg(ssGSEA_old) – ssGSEA_young)*). We used the wilcox. test() function in R to calculate the Wilcoxon *p*‐value and used the *p*.adjust() function to find the adjusted *p*‐values using the false discovery rate method (FDR) for all regulons.

### 2.5 Cell–cell communication analysis

We used the CellChat R package (version 1.1.3; Jin et al., 2021) to infer and analyze intercellular communication via ligand‐receptor integrations. CellChat algorithm (Jin et al., 2021) exploits the built‐in information of a signaling molecule interaction database and infers a quantitative detection of intercellular communication networks. Conserved and context‐specific signaling pathways were identified by comparing the overall information flow at different conditions (age group). The information flow of a signaling pathway represents the overall communication probability among all pairs of cell types in the ligand‐receptor network. Only signaling pathways with a difference of scaled information flow >10 and P value <0.05 were selected for visualization.

### 2.6 Cell culture

Primary mouse embryonic fibroblasts (MEF) were isolated from wild‐type C57BL/6J mice as previously described and cultured in DMEM (4.5 g/L glucose) supplemented with 10% FBS for different days at 37°C with 5% CO_2_.

### 2.7 pBabe‐LEF1 construct and retroviral infection

pBabe‐puro LEF1 (the long isoform) was a gift from Joan Massague (Addgene plasmid # 27023; http://n2t.net/addgene:27023; RRID:Addgene_27,023). Retroviruses were produced by transiently transfecting HEK293T cells with a mixture of pBabe/pBabe‐LEF1 and pCL‐ampho plasmids. Seventy‐two hours after transfection, retroviruses were collected and used to infect day 1MEFs with a MOI of 3 for 48 h in the presence of 8 μg/mL polybrene.

### 2.8 Immunoblots

Whole cell lysates were isolated from MEFs with RIPA buffer (150 Mm NaCl, 1% Triton X‐100, 0.5% sodium deoxycholate, 0.1% SDS, and 50 mM Tri (pH 7.4) with protease and phosphatase inhibitors). Protein concentrations were determined by Bradford assays (Bio‐Rad), and aliquots were snap‐frozen in liquid nitrogen and stored at −80°C until usage. Immunoblot analyses were performed as described previously (Zhu et al., 2015). Briefly, 25 μg proteins separated by 4%–20% gradient SDS‐PAGE gels (Bio‐Rad) were transferred to nitrocellulose membranes, blocked in TBST buffer supplemented with 5% bovine serum albumin (BSA) or 5% fat‐free milk and incubated overnight with primary anti‐LEF1 antibody (Cell Signaling, #2230), anti‐p16 antibody (ThermoFisher, # PA5‐20379) at 4°C overnight. Blots were incubated with an appropriate secondary antibody coupled to horseradish peroxidase at room temperature for 1 h and reacted with ECL reagents per the manufacturer's (Thermo Scientific) suggestion and detected by Biorad ChemiDoc MP Imaging System.

Human lung tissues were homogenized and lysed in RIPA buffer. Proteins were quantified by Pierce™ BCA Protein Assay (Thermo Scientific). Equal amounts of proteins (50 μg) from cell preparations were separated by sodium dodecyl sulfate‐polyacrylamide gel electrophoresis (SDS‐PAGE) and electrotransferred to a PVDF membrane (Bio‐Rad) using a Trans‐Blot Turbo™ transfer system (Bio‐Rad). After transfer, membranes were washed in TBS‐T (10 mM Tris, pH 8.0, 150 mM NaCl, 0.05% Tween 20), and blocked with TBS‐T supplemented with 5% non‐fat dry milk (Dry Powder Milk, RPI) for 1 h at RT. Then, membranes were incubated overnight at 4°C with different primary antibodies in TBS‐T against LEF‐1 (1:500, Antibody# 2230, Cell Signaling Technology), and β‐actin (1:30,000, A3854, Sigma‐Aldrich). Next day, membranes were washed, and incubated with horseradish peroxide‐conjugated secondary antibody (1:2000, Antibody#7074, Cell Signaling Technology) for 1 h at RT. Following additional wash steps with TBS‐T, membranes were treated with Clarity™ western ECL substrate (Bio‐Rad). Quantification was performed by measurement of signal intensity with Image J software (National Institute of Health, Bethesda, MD, USA). Statistical analysis was performed using the pairwise non‐parametric Mann–Whitney test from the GraphPad Prism software.

### 2.9 qRT‐PCR

Total mRNA was isolated from MEFs with PureLink RNA mini kit (Life Technologies) with additional on‐column DNase digestion step to remove genomic DNA per the manufacturer's instructions. Reverse transcription was carried out using 5 μg of RNA using Superscript III (Life Technologies) per the manufacturer's instructions. For gene expression analyses, cDNA samples were diluted 1/30‐fold (for all other genes except for 18sRNA) and 1/900‐fold (for 18sRNA). qPCR was performed using the SYBR green system with sequence‐specific primers (Table S2). All data were analyzed with 18S or β‐actin as the endogenous control and all amplicons span introns. Data were analyzed and presented with GraphPad Prism software. Plots show individual data points and bars at the mean ± SEM. One‐tailed *t*‐tests were used to compare means between groups, with significance set at *p* < 0.05.

### 2.10 Senescence‐associated β‐galactosidase (SA‐β‐gal) assay

Cells were washed twice with phosphate‐buffered saline (PBS; pH 7.2), fixed with 0.5% glutaraldehyde in PBS and washed in PBS supplemented with 1 mM MgCl_2_. Cells were stained at 37°C in X‐Gal solution (1 mg/mL X‐Gal, 0.12 mM K3Fe[CN]6, 0.12 mM K4Fe[CN]6, 1 mM MgCl_2_ in PBS at pH 6.0). The staining was performed for 24 h at 37°C.

## 3 RESULTS

### 3.1 Age‐related gene expression changes are cell‐ and species‐specific

**FIGURE 1:**
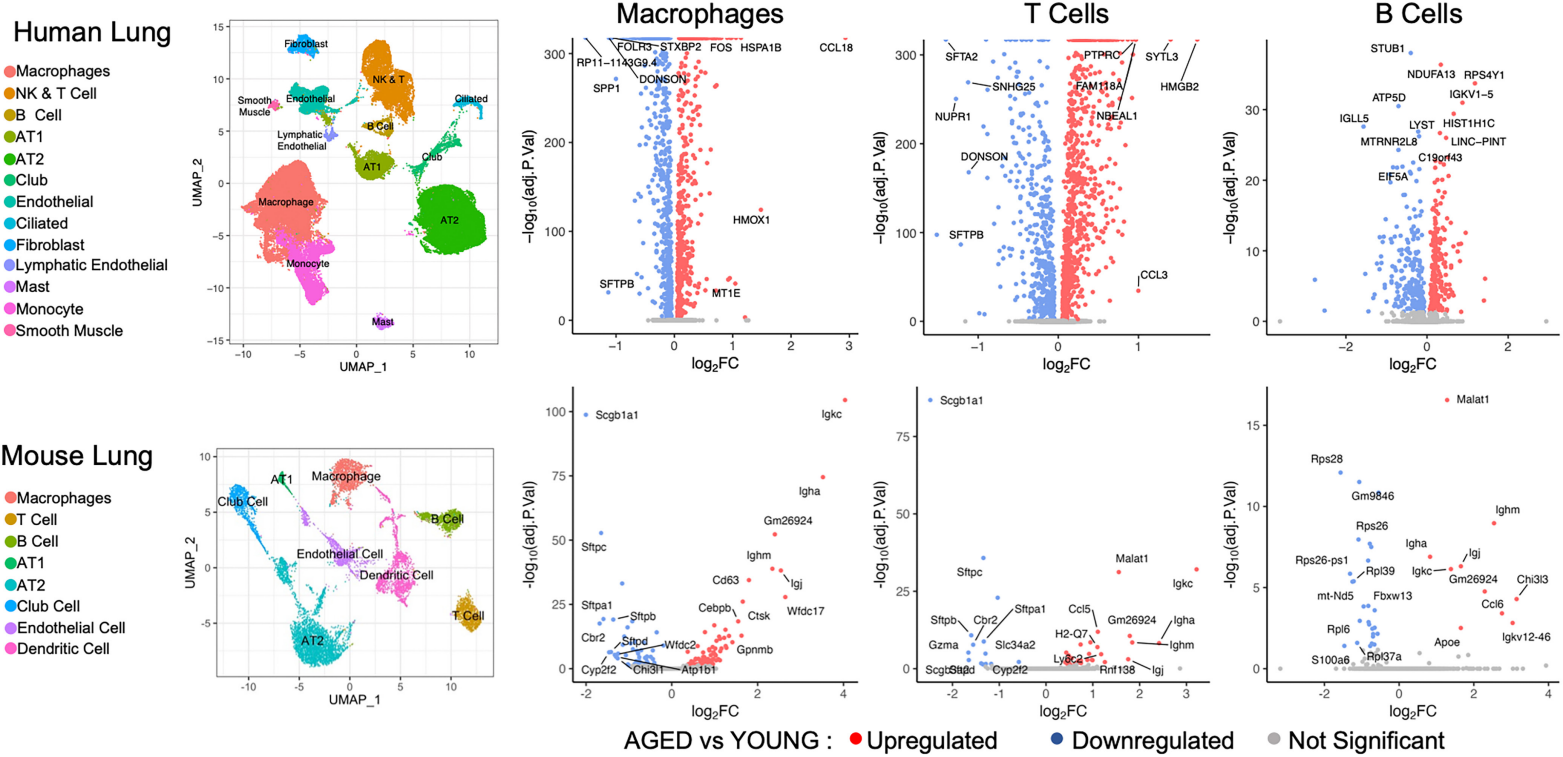
Identification of differentially expressed genes in the human and mouse lung datasets. UMAP plots show the main clusters in the human and mouse lung datasets. Volcano plots show differentially expressed genes in human and murine lung Macrophages, T Cells, and B cells. Significance threshold is set at adj.*p* < 0.05. The names of the top 10 highly upregulated/downregulated genes are shown.

To uncover important drivers of aging, we examined publicly available human and murine scRNA‐seq lung datasets. The datasets included young and aged healthy donors. As a first step, we clustered the different cell types (Figure 1, UMAPs and Figure S1) and we identified seven cell types that were found in both datasets (i.e., Macrophages, T Cells, B Cells, AT1, AT2, Club Cells, and Endothelial Cells). The mouse lung dataset included a separate cluster of Dendritic Cells whereas the human lung dataset included separate clusters for Monocyte, Fibroblast, Ciliated, Mast, Lymphatic Endothelial, and Smooth Muscle Cells. Subsequently, we performed differential gene expression (DGE) analysis between samples from old and young donors, focusing on immune cell types that were well represented in both datasets. Further analysis showed that each cell type displayed significant age‐related changes in the expression of multiple genes but, only 24 genes were found in all immune cell types (Figure 1, top volcano plots): NDUFA13, RPS4Y1, HNRNPC, ANKRD28, LRRFIP1, FKBP5, TSC22D3, KLF6, FOS, NBEAL1, CD44*ATP5D, EIF5A, CST3, S100A11, GRN, FIS1, S100A9, SNHG8, NDUFA3, ATP5I, NDUFB1, POLR2L and IFITM3*. Similarly (Figure 1, bottom volcano plots), we found eight genes significantly changing with age across macrophages, T cells, and B cells in the murine dataset: *Scgb1a1*, *Igkc*, *Malat1*, *mt‐Rnr2*, *Gm26924*, *Ighm*, *Igha*, *Igj*. Interestingly, none of the differentially expressed genes was common for both species.

### 3.2 Cell–cell communication analysis revealed dysregulation of age‐related cellular pathways

**FIGURE 2:**
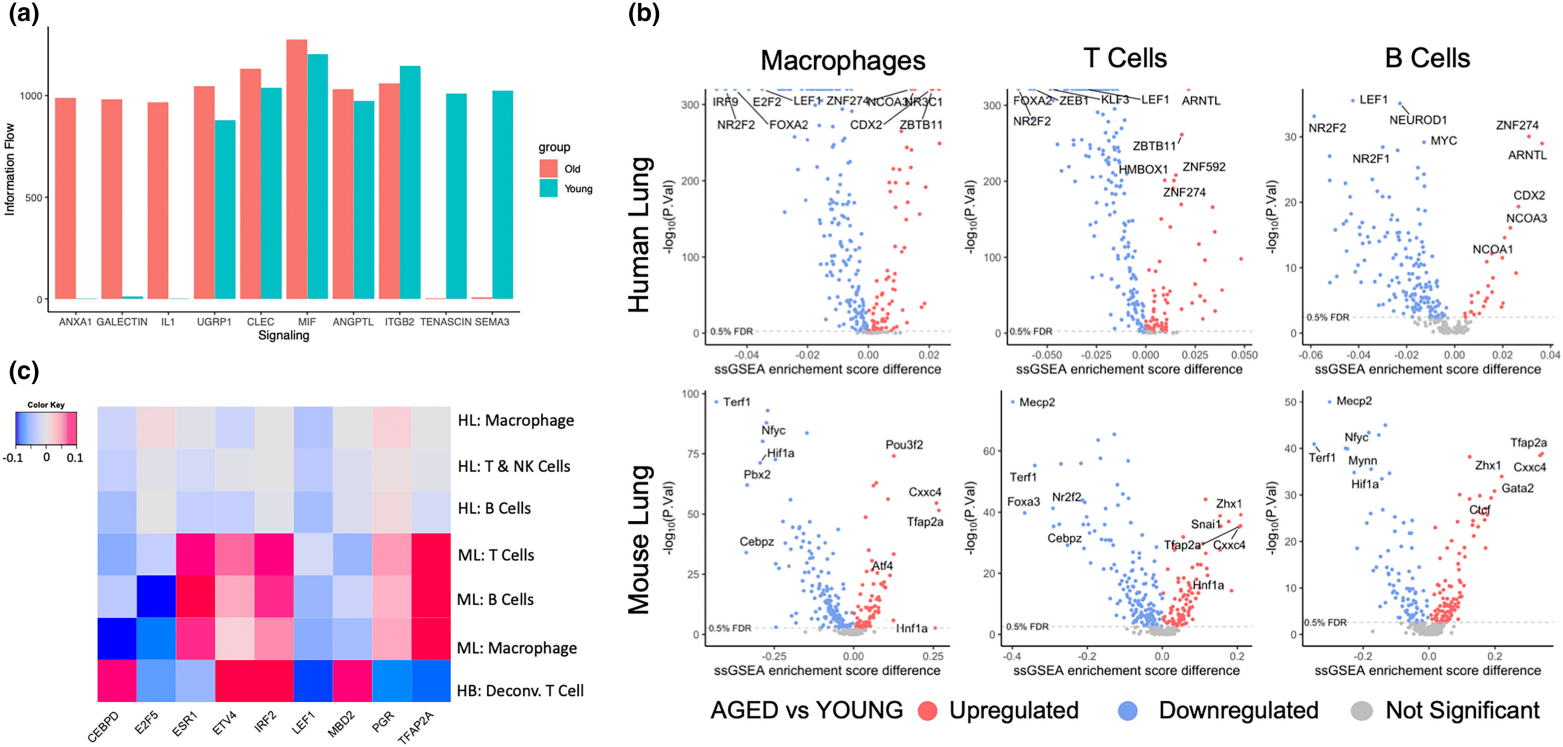
Age‐related differences in the activity of cell communication networks and regulons. (a) Information Flow of CellChat inferred signaling ligands in young and aged human lungs. (b) Calculation of the differential regulon activity (ssGSEA score difference) in human lung Macrophages, T Cells, B cells (TOP), and murine Macrophages, T Cells, B Cells (BOTTOM). (c) Identification of common regulons between human (lung and blood) and mouse (lung) cells. Heatmap shows the difference between the average regulon ssGSEA scores in old versus young samples, with FDR <0.1 in all cell types. HB, human blood; HL, human lung; ML, mouse lung.

We used CellChat to generate an information flow for each signaling pathway that was identified in lung cells from both young and aged donors, and our analysis revealed age‐related changes in several of these pathways. In aged cells, we observed a dramatic increase in ANXA1 (annexin A1), LGALS9 (galectin 9), and IL1 (interleukin 1) network activity while TENASCIN and SEMA3 (semaphorin 3) networks suffered a significant decrease. To a lesser extent, uteroglobin‐related protein 1 (UGRP1), C‐type lectin domain (CLEC), macrophage migration inhibitory factor (MIF), and angiopoietin like (ANGPTL) network activities were also increased in aged cells while ITGB2 decreased (Figure 2a).

### 3.3 LEF1 regulon is consistently found less active in aged subjects

Focusing on the immune cells that were used in our DEG analysis, we compared regulon activity between young and aged donors. To this end, we calculated ssGSEA scores for all regulons included in the DoRothEA gene regulatory network and found that 141 regulons were significantly changed with age in humans (Wilcoxon *p* < 0.05) (Figure 2b, top) while 132 regulons were significantly changed in mice (Figure 2b, bottom). Based on differential regulon activity, we identified 70 regulons showing a significant age‐related change in both human and murine lung cells. To narrow down the search for factors that could have a more universal role in aging, we analyzed data from the COPDGene study. We calculated the differential ssGSEA scores of the blood samples from old and young healthy donors, compared them to our previous results, and found nine common regulons (CEBPD, E2F5, ESR1, ETV4, IRF2, LEF1, MBD2, PGR, and TFAP2A) that were significantly changed in our human lung, mouse lung, and human blood samples. Of these nine, only *LEF1* regulon activity was consistently decreased with age in all datasets (Figure 2c).

### 3.4 LEF1 expression is dysregulated during cellular senescence

**FIGURE 3:**
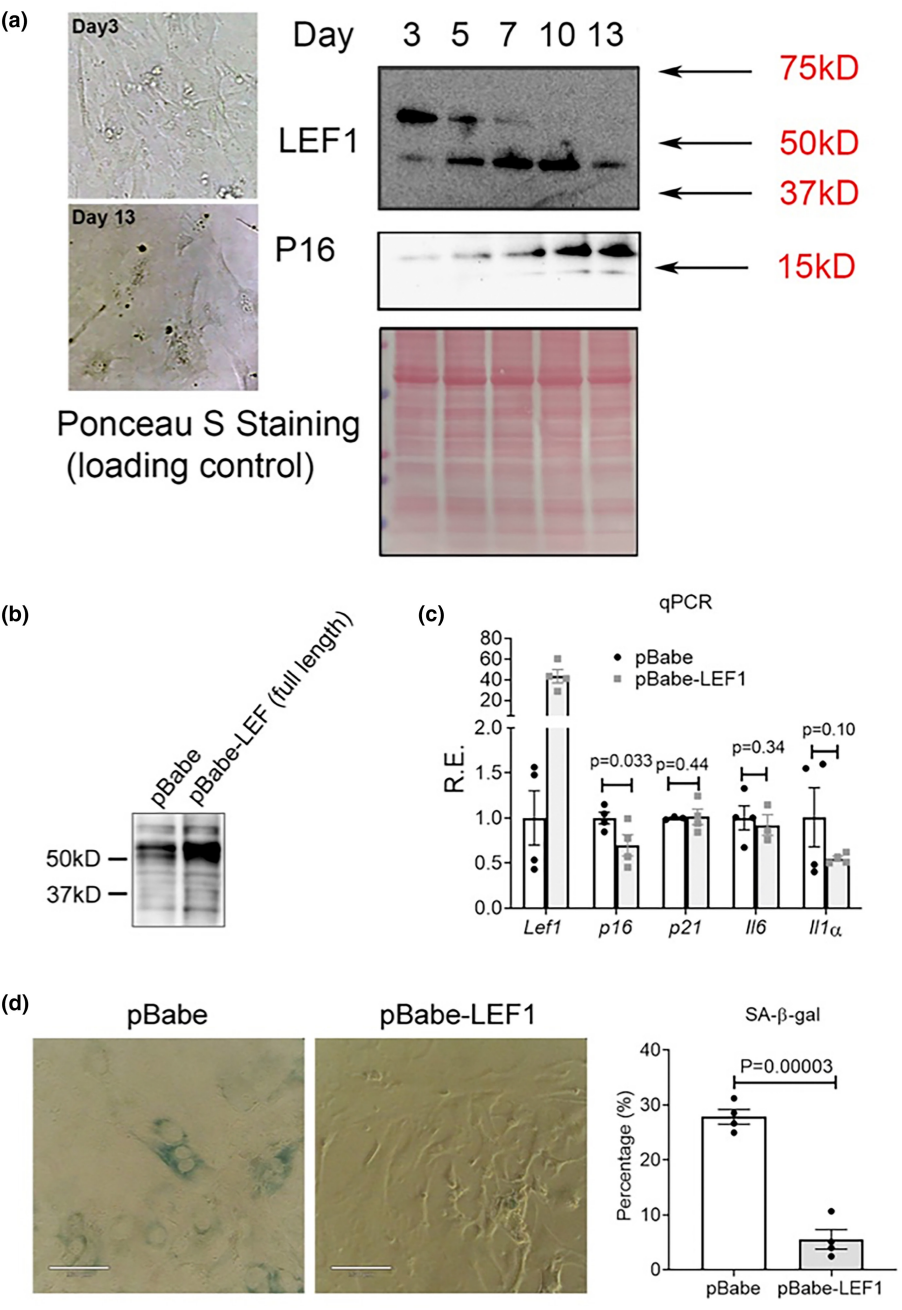
LEF1 is implicated in cellular senescence. (a) Representative images of the morphology of MEFs at Day 3 and Day 13 after culturing. Immunoblot detection of p16 and LEF1 expression at different days. Ponceau S staining is provided as a loading control. (b) Immunoblot detection of LEF1 in cells expressing pBabe‐empty vector or pBabe‐LEF1 (long isoform) construct. (c) qPCR analysis of the expression of *Lef1* and four common senescence markers at day 9 (8 days post retroviral infection) in MEFs. Results are shown as relative expression changes compared to cells transfected with the empty vector. (d) Representative images (left) and quantification (right) of SA‐β‐Gal staining in control or LEF1‐overexpressing MEFs at day 9 (8 days post retroviral infection).

Since cellular senescence is a major hallmark of aging (López‐Otín et al., 2023), we next examined whether LEF1 expression also changes during cellular senescence. Primary mouse embryonic fibroblasts undergo spontaneous cellular senescence in culture, characterized by flattened cell morphology, increased expression of senescence marker CDKN2A/p16INK4a/p16, and increased senescence‐associated β‐galactosidase (SA‐β‐Gal) activity (Figure 3a,d; Guan et al., 2020; Manning & Kumar, 2010). Interestingly, we detected two LEF1 isoforms that were regulated in a completely anti‐parallel fashion. While the longer LEF1 isoform (WT‐LEF1) (~60 kD) was decreasing to undetectable levels during cellular senescence, the shorter isoform (~40 kD) gradually increased (Figure 3a). The size of the shorter protein is consistent with the size of a dominant‐negative LEF1 isoform, lacking the N‐terminus β‐catenin‐binding domain (Hovanes et al., 2001).

To investigate whether the decrease in the longer LEF1 isoform contributes to cellular senescence, we ectopically expressed the longer LEF1 isoform in MEFs at Day 1 and quantify its impact on cellular senescence progression (Figure 3b). The ectopic longer LEF1 isoform expression resulted in the significant downregulation of p16 expression but did not affect some other of the commonly cellular senescence markers, although *IL1α* expression was marginally reduced (Figure 3c). Nevertheless, staining with β‐galactosidase showed a significant decrease of more than threefold, in the number of senescent cells upon the ectopic expression of longer isoform LEF1 (Figure 3d). Together, these results demonstrate a possible role of the longer isoform of LEF1 in partially attenuating cellular senescence.

### 3.5 LEF1 regulon activity in IPF lungs

**FIGURE 4:**
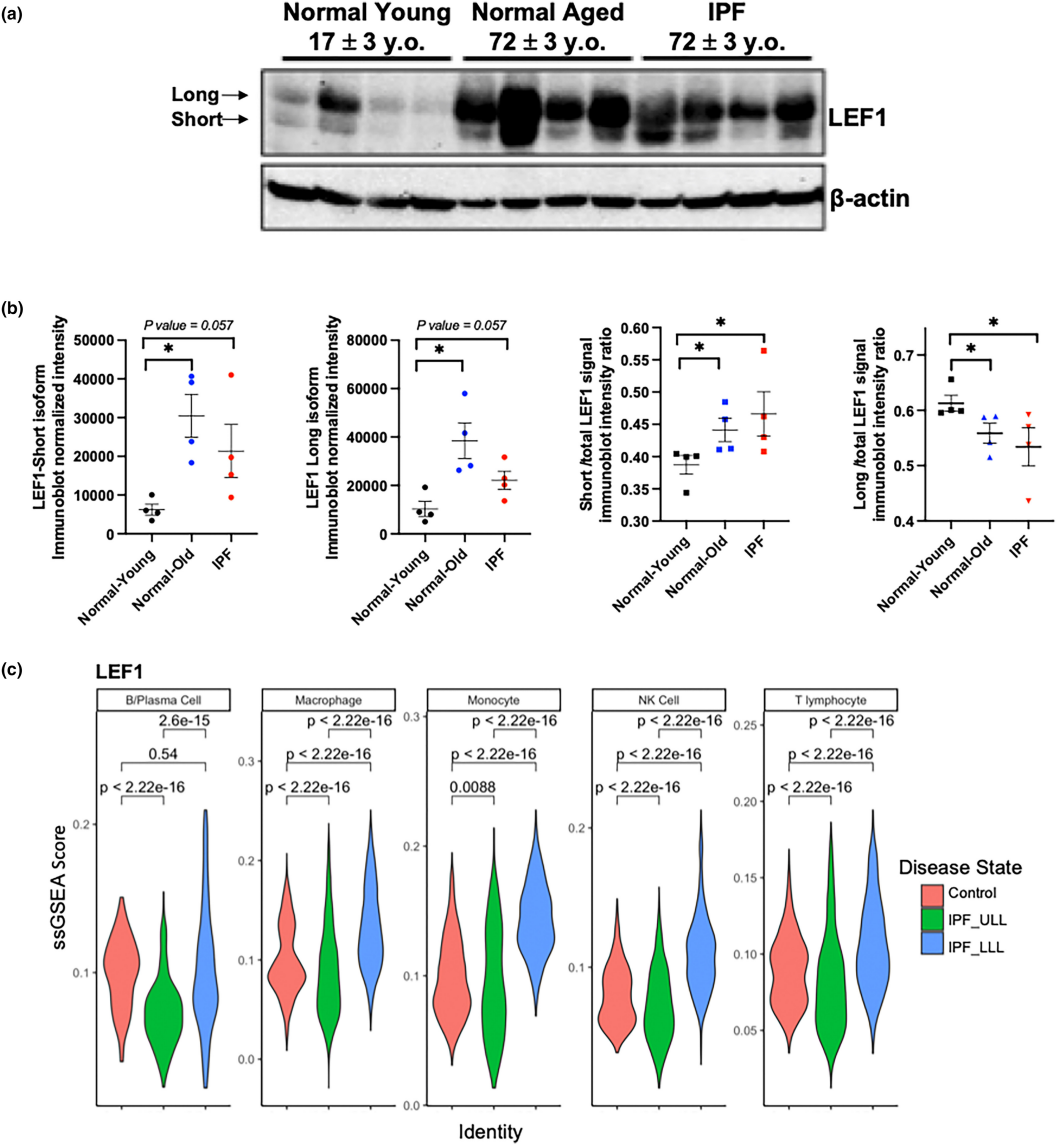
LEF1 expression in human lung tissue lysates. (a) LEF1 immunoblot of four normal healthy young, four healthy old, and four IPF human lung tissue lysates. (b) Quantification of LEF1 isoforms using β‐actin used as a loading control. Data represent the mean value ± SEM (*n* = 4 samples evaluated per group). *p < 0.03, pairwise non‐parametric Mann–Whitney test. (c) Violin plot graphs showing LEF1 ssGSEA scores in human lung immune cells. IPF, idiopathic pulmonary fibrosis; IPF‐LLL, lower lung lobe; IPF‐ULL, upper lung lobe.

Due to the important role aging and cellular senescence play in the pathophysiology of idiopathic pulmonary fibrosis (IPF) (Kellogg et al., 2021), we checked LEF1 expression in lung tissue from Normal/Healthy and IPF lung donors (Figure 4a,b). Since IPF patients tend to be older and their phenotype is shaped by both age and disease, we included in our analysis aged‐matched healthy lung donors. Immunoblot assays of lung tissue showed an overall increase in LEF1 protein in older healthy and IPF donors. Quantification of the two isoforms in all donor groups revealed a higher increase in the short isoform (FC = 4.9) than the long isoform (FC = 3.7) in healthy old lungs compared to healthy young lungs. Although less significant, the same trend was observed in IPF lungs where the short and long isoforms showed a 3.4‐ versus a 2.1‐fold change increase respectively. When the relative abundance of both isoforms was evaluated as the ratio to the total LEF1 signal, we observed a significant increase in the relative amount of the short LEF1 isoform (Figure 4b) coupled with a significant decrease in the relative amount of the long LEF1 isoform.

When we examined the activity of LEF1 regulon in lung immune cells from upper lung lobes (IPF_ULL), which typically show very little histological change, and lower lung lobes (IPF_LLL), which are greatly affected by the disease (Jia, Rosas, et al., 2023; Morse et al., 2019), we found that overall, the activity was decreased in the upper lobe of IPF lungs, supporting the presence of aging/senescent cells. Interestingly, immune cells from the lower and highly fibrotic IPF lung lobes show increased LEF1 regulon activity, suggesting a more complex LEF1 regulatory pathway in advanced fibrosis that is not limited to cellular senescence **(**Figure 4c **)**.

## 4 DISCUSSION

In this work, we investigate the important topic of mechanisms of aging and aging‐related pathologies. The study of such multiparametric phenomena could benefit from computational approaches that take advantage of the increasing number of available datasets, which include samples from a wide spread of age groups. Although many of these studies do not directly question the effects of aging, their control samples constitute a really underexplored valuable resource. In our analyses, we utilized and compared data from three different sources: scRNAseq data from an IPF‐related study, blood bulk RNAseq data from healthy smoker population of COPDGene®, and scRNAseq data from an aging‐related mouse study.

Our aim was to identify key pathways that are affected throughout aging in all immune cells under investigation. In accordance with a previous study (Barth et al., 2019), the conservation of differentially expressed genes across tissues and species was limited. Nevertheless, when we focused on alterations in intercellular communication, ANXA1, LGALS9, and IL1 signaling pathways showed significant activation with age while TENASCIN and SEMA3 showed significant decline. ANXA1 has a known anti‐inflammatory role (Jia et al., 2013; Rubinstein et al., 2019) and the observed activation could indicate a response to an age‐related inflammatory state (inflammaging; Franceschi et al., 2018). The IL1 cytokine signaling has been implicated in the inflammation of several tissues including the lung (Borthwick, 2016) as well as in promoting cellular senescence (Maier et al., 1990). GALECTIN9 has emerged as a multipotent immunomodulatory molecule (John & Mishra, 2016) and a biomarker of disease severity (Moar & Tandon, 2021). The decreased TENASCIN signaling, another diverse immune response mechanism (Midwood & Orend, 2009), could associate with the age‐related decline of the immune system (Weyand & Goronzy, 2016). SEMA3 signaling has been implicated in immune cell migration, cooperation and deactivation (Kiseleva & Rutto, 2022) and its decreased activity in older donors could be associated with compromised immune response as well as prolonged inflammation.

Further analysis based on the activity of regulatory modules (regulons), in all cell types and species, resulted in ubiquitous age‐related differences in 13 regulons. LEF1 regulon was the only regulon with a consistent pattern of age‐dependent activity in both human and murine immune cells. LEF1 is a TF expressed in B and T cells (Elyahu et al., 2019; Milatovich et al., 1991). It is implicated in various cancers where its overexpression is associated with poor prognosis (Erdfelder et al., 2010; Eskandari et al., 2018). LEF1 interacts with β‐catenin (Behrens et al., 1996) and SMAD2‐3 (Labbé et al., 2000), key molecules in the Wnt and TGF‐β pathways respectively. Interestingly, Wnt and TGF‐β signaling pathways can independently or synergistically regulate LEF1 targets (Labbé et al., 2000). Both pathways are dysregulated in cellular senescence and aging (Hu et al., 2020; Tominaga & Suzuki, 2019) and LEF1 is found to be downregulated in certain aged tissues (Barth et al., 2019). Moreover, haplosufficiency in the LEF1 locus has been associated with low bone mass (Noh et al., 2009), a common feature of aging. While the function of long LEF1 isoform and the significance of LEF1 expression levels have been studied, the fluctuation of the smaller isoform, lacking the β‐catenin binding domain, has less clear consequences. Some studies suggest that the small isoform acts as a dominant negative variant inhibiting β‐catenin binding and consequently promoter activation (Hovanes et al., 2001), while other studies support a more complex role where the short isoform has distinct targets and biological functions (Edmaier et al., 2013). Notably, we observed that the overall LEF1 regulon activity invariably decreased with age but the expression profiles of several LEF1 target genes did not change consistently among the various cell types and across organisms, indicating that downstream signals may be diverse and cell‐type specific. This could explain why many studies were unable to detect significant conservation in DGE profiles of the immune cells across species; and it is consistent with previous reports that aging could have a subtle transcriptional signature depending on the tissue type (Barth et al., 2019), further hindering the detection of ubiquitous aging‐driving factors.

Immunodetection of LEF1 in mouse embryonic fibroblasts revealed a senescence‐associated dysregulation of two distinct isoforms. The longer isoform showed a steady decrease with increased replicative senescence (indicated by increased p16 expression, a known senescence marker; Guan et al., 2020), while the short isoform was gradually upregulated. Ectopic expression of the longer isoform prevented replicative senescence, supporting the role of the full‐length LEF1 in cell proliferation (Hao et al., 2019). Previous work had shown that LEF1 can inhibit p16 expression through promoter binding (Delmas et al., 2007). In view of the key role of cellular senescence in IPF (De Man et al., 2023; Schafer et al., 2017), we measured the protein levels of both LEF1 isoforms in tissue lung samples from healthy and IPF donors. It is important to note that lung tissue in general and IPF fibrotic tissue in particular present a challenging material for analysis due to the high cell type heterogeneity and the spatial diversity of the disease progression. Whereas lung immune cells from older subjects show a less active LEF1 regulon, suggesting a possible decrease in the long LEF1 isoform expression, protein levels were increased in both healthy old and IPF lung tissue. Interestingly, in old and IPF lungs, the relative amount of the long isoform decreased while that of the short isoform increased, which is reminiscent of the LEF1 dysregulation pattern in our senescent MEFs. Unsurprisingly, the LEF1 regulon activity was decreased in the upper lung lobes of IPF patients in agreement with our findings in old healthy lungs. This suggests that immune cells from older lungs as well as normal‐looking areas from IPF lungs could undergo a LEF1/senescence‐related decline. LEF1 regulon activity in the immune cells of the highly fibrotic lower lobes appears elevated, suggesting a diverse and rather more proliferative state in these cells. Overall, our results show that LEF1 levels in lung tissue are mainly defined by donor age, but specifically in immune cells LEF1 may orchestrate two diverse responses depending on the disease state.

Although we limited the computational analysis to macrophages, T cells and B cells, we were able to identify LEF1 as a common age‐dependent regulator. Our findings indicate that LEF1 protects from cellular senescence, but due to its pleiotropic nature, we cannot exclude that could promote aging through different pathways that counteract its anti‐senescence role. Taking into consideration the key role of immune cells in aging and disease (Franceschi et al., 2018; Fulop et al., 2018; Jia, Agudelo Garcia, et al., 2023), our results offer a significant contribution to the field. Without a doubt, more research is required to determine whether LEF1 dysregulation is intrinsic to the aging process, leading to increased vulnerability to cellular malfunction and disease. Clearly though, LEF1 dysregulation, and by extension, the dysregulation of its downstream cellular factors and pathways, appears to be cell‐specific, which dictates more targeted and precise approaches for future aging studies.

## AUTHOR CONTRIBUTIONS

Panayiotis V. Benos and Maria G. Kapetanaki designed the study with the help of Minxue Jia and Khaled Sayed. Minxue Jia and Khaled Sayed performed all computational analyses. William Dion, Lorena Rosas, and Saad Irfan performed the experiments under the supervision of Mauricio Rojas and Bokai Zhu. Minxue Jia, Khaled Sayed, Maria G. Kapetanaki, and Panayiotis V. Benos wrote the paper with input from Eleanor Valenzi, Ana L. Mora, Robert A. Lafyatis, Mauricio Rojas, and Bokai Zhu. All authors have read and approved this manuscript.

## FUNDING INFORMATION

This work was partly supported by the following grants from the National Institutes of Health (NIH): U01HL145550 (ALM, RAL, MR, PVB); R01HL157879, R01HL127349, R01AA028436 (PVB); DP2GM140924 (BZ). The COPDGene® study (NCT00608764) was supported by NHLBI U01HL089897, U01HL089856, and by the COPD Foundation through contributions made to an Industry Advisory Committee that has included AstraZeneca, Bayer Pharmaceuticals, Boehringer Ingelheim, Genentech, GlaxoSmithKline, Novartis, Pfizer, and Sunovion.

## CONFLICT OF INTEREST STATEMENT

RAL has received funding from Corbus, Formation, Moderna, Regeneron, Astra Zeneca, Pfizer; and consulting fees from Pfizer, Bristol Myers Squibb, Boehringer‐Ingleheim, Formation, Sanofi, Boehringer‐Mannheim, Merck, Genentech/Roche, Biogen.

## Supporting information


Data S1:

Figure S1.

## Data Availability

Data were retrieved from public databases (GEO GSE128033, GSE122960, GSE124872, GSE158699).
