# Supplementary material for: LEF1 isoforms regulate cellular senescence and aging
Source: Aging Cell. 2023 Nov 13;22(12):e14024. doi: 10.1111/acel.14024 (PMC10726832; doi:10.1111/acel.14024)
Supplement: Supplementary file 1 — Data S1: [file ACEL-22-e14024-s001.docx]

**LEF1 isoforms regulate cellular senescence and aging**

**SUPPLEMENTARY MATERIALS.**

**Supplementary Table S1**. Samples used for cell type identification. Samples Donor 2 and Donor 4 were excluded from young vs aged comparisons due to recorded or suspected previous smoking status.

| **Sample ID** | **Age** | **Gender** | **Age group** | **Smoking Status** | **Included in analyses** | **GEO** |
| --- | --- | --- | --- | --- | --- | --- |
| Donor 1 | 63 | Female | AGED | Never | Y | GSE122960 |
| Donor 2 | 55 | Male | AGED | Former |  | GSE122960 |
| Donor 4 | 57 | Female | AGED | Potential Former |  | GSE122960 |
| Donor 6 | 22 | Female | YOUNG | Never | Y | GSE122960 |
| Donor 8 | 21 | Male | YOUNG | Never | Y | GSE122960 |
| SC56 | 57 | Female | AGED | None | Y | GSE128033 |
| SC59 | 18 | Male | YOUNG | None | Y | GSE128033 |
| SC155 | 23 | Female | YOUNG | None | Y | GSE128033 |
| SC156 | 23 | Female | YOUNG | None | Y | GSE128033 |
| SC45 | 55 | Male | AGED | NA | Y | GSE128033 |

**Supplementary Table S2**. qRT-PCR primer list

| Mouse 18s RNA FOR primer | ctcaacacgggaaacctcac |
| --- | --- |
| Mouse 18s RNA REV primer | cgctccaccaactaagaacg |
| Mouse β-actin FOR primer | aaggccaaccgtgaaaagat |
| Mouse β-actin REV primer | gtggtacgaccagaggcatac |
| Mouse/Human Lef1 FOR primer | cggaactctgcgcc |
| Mouse/Human Lef1 REV primer | ctggccttgtcgtgg |
| Mouse p16 (Cdkn2a) FOR primer | gggttttcttggtgaagttcg |
| Mouse p16 (Cdkn2a) REV primer | ttgcccatcatcatcacct |
| Mouse p21 (Cdkn1a) FOR primer | tccacagcgatatccagaca |
| Mouse p21 (Cdkn1a) REV primer | ggacatcaccaggattggac |
| Mouse Il6 FOR primer | acaaagccagagtccttcaga |
| Mouse Il6 REV primer | tggtccttagccactccttc |
| Mouse Il1α FOR primer | gcttgagtcggcaaagaaatc |
| Mouse Il1α REV primer | gagagatggtcaatggcagaa |
