# Supplementary material for: LEF1 isoforms regulate cellular senescence and aging
Source: Aging Cell. 2023 Nov 13;22(12):e14024. doi: 10.1111/acel.14024 (PMC10726832; doi:10.1111/acel.14024)

LEF1 alternative transcription regulation affects cellular senescence and aging

SUPPLEMENTARY FIGURES

Supplementary Figure S1

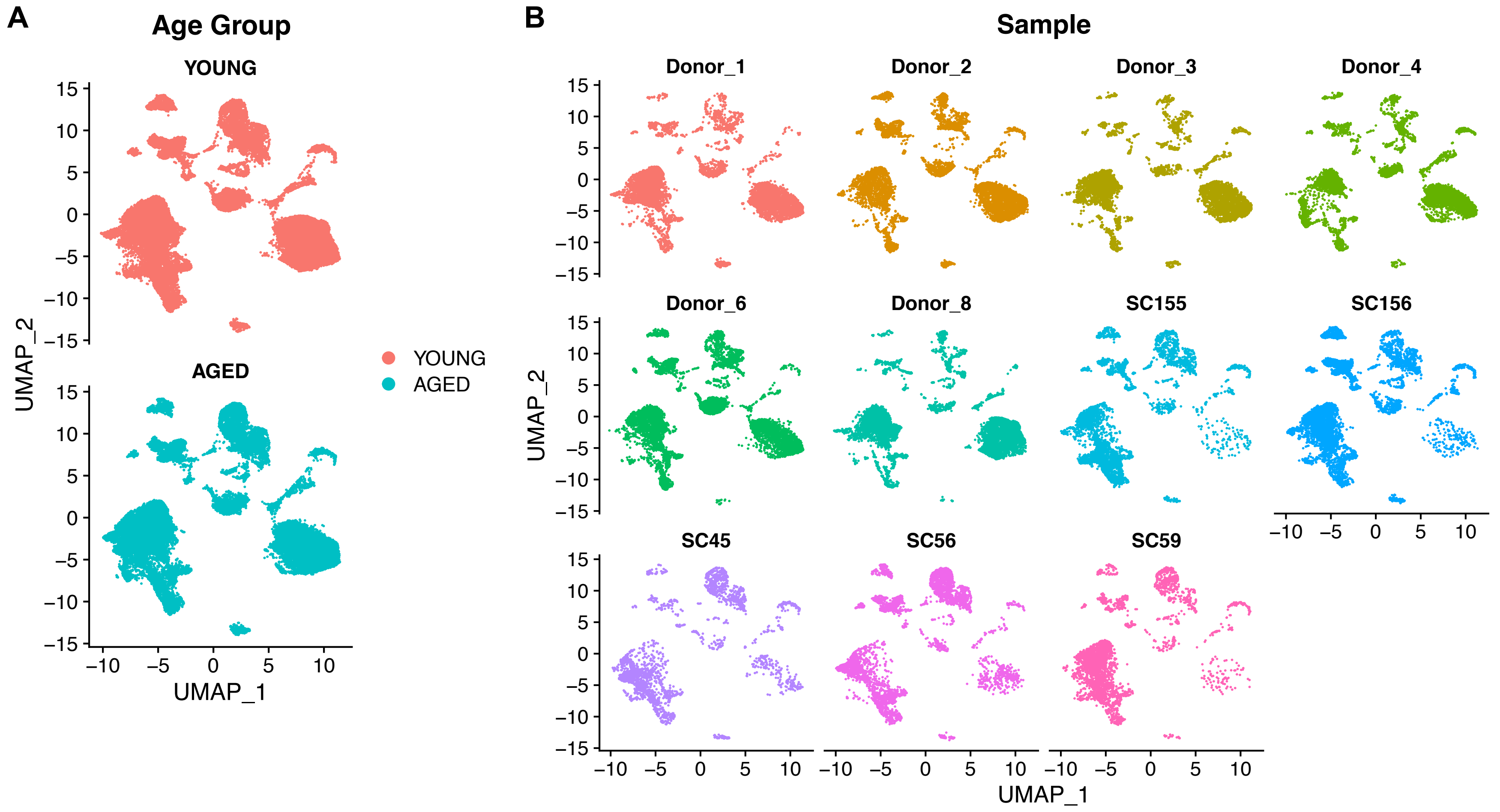

Supplementary Figure S2

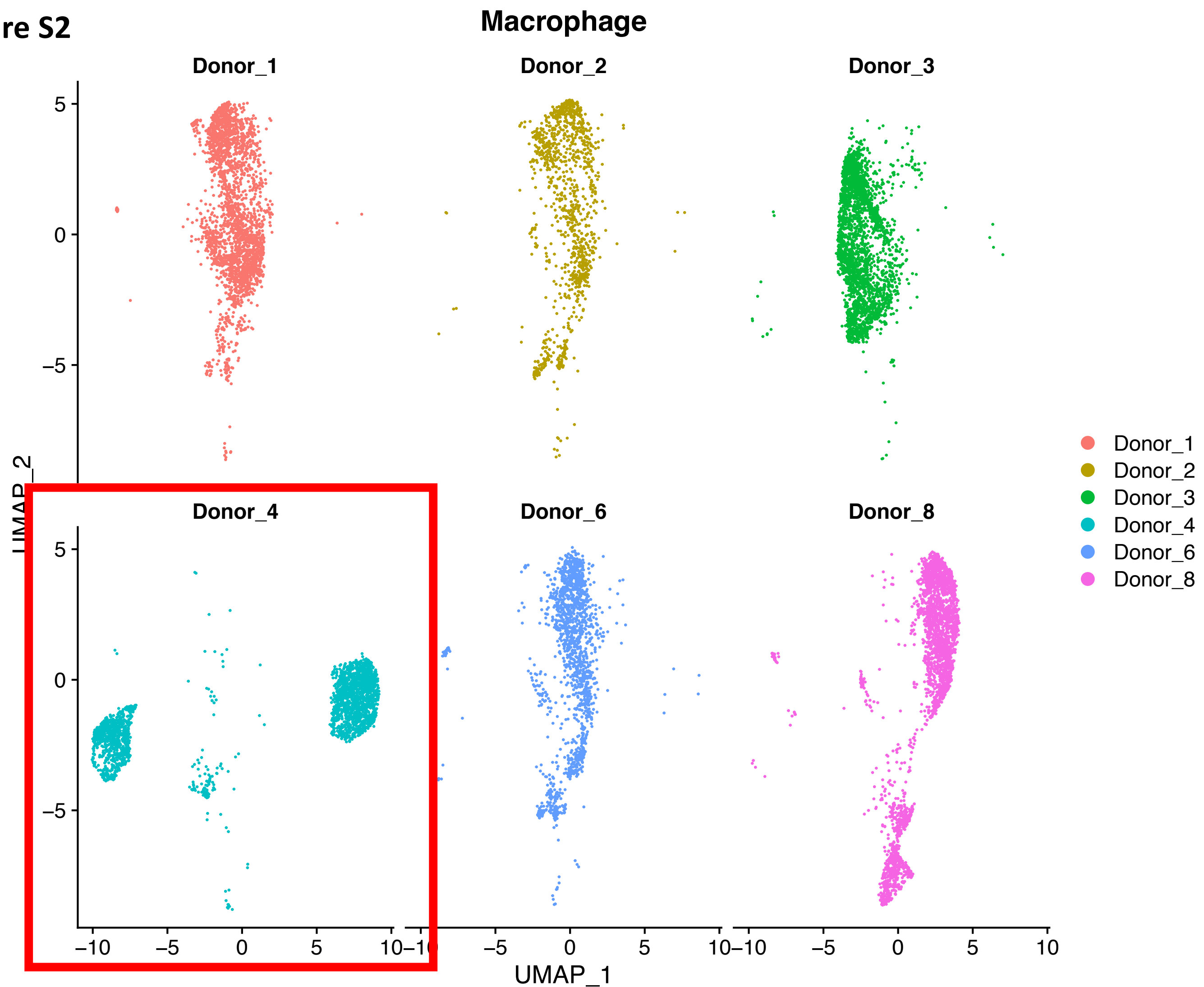

Supplementary Figure S3

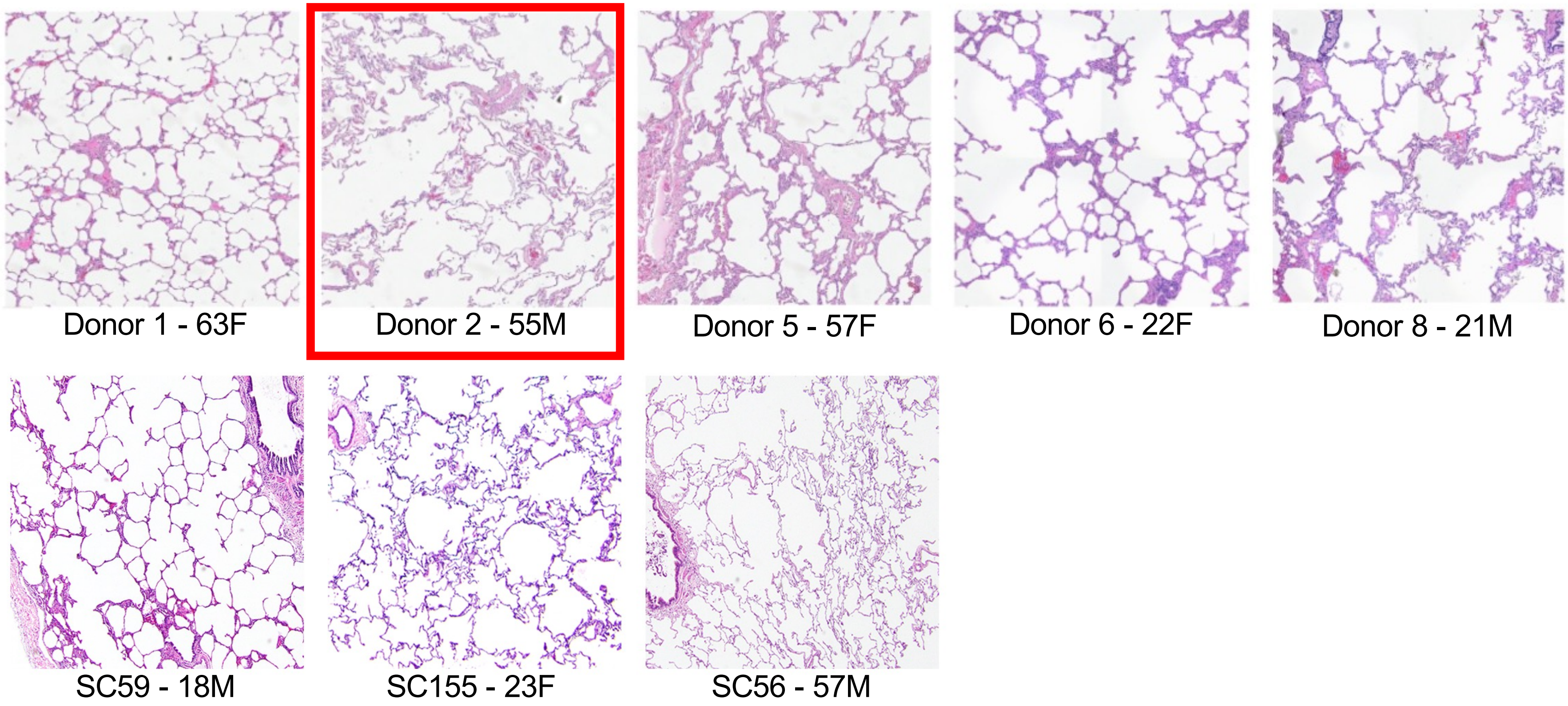

Supplement: Supplementary file 2 — Figure S1. [file ACEL-22-e14024-s002.pdf]
